# Cholestasis Reveals Severe Cortisol Deficiency in Neonatal Pituitary Stalk Interruption Syndrome

**DOI:** 10.1371/journal.pone.0147750

**Published:** 2016-02-01

**Authors:** Francois-Xavier Mauvais, Emmanuel Gonzales, Anne Davit-Spraul, Emmanuel Jacquemin, Raja Brauner

**Affiliations:** 1 Pediatric Hepatology and Liver Transplantation Unit, Reference centre for pediatric liver diseases–DHU Hepatinov, Hôpital Bicêtre, Assistance Publique—Hôpitaux de Paris, Le Kremlin Bicêtre, France; 2 Institut National de la Santé et de la Recherche Médicale Unité 1151 et Centre National de la Recherche Scientifique, UMR8253, Paris, France; 3 Université Paris Descartes, Sorbonne Paris Cité, Faculté de médecine, Paris, France; 4 Institut National de la Santé et de la Recherche Médicale UMR-S 1174, Orsay, France; 5 Université Paris-Sud 11, Faculté de médecine, Le Kremlin Bicêtre, France; 6 Biochemistry laboratory, Hôpital Bicêtre, Assistance Publique—Hôpitaux de Paris, Le Kremlin-Bicêtre, France; 7 Pediatric Endocrinology Unit, Fondation Ophtalmologique Adolphe de Rothschild, Paris, France; Innsbruck Medical University, AUSTRIA

## Abstract

**Objectives:**

Cholestasis has been reported during the course of congenital hypothalamic-pituitary deficiency, but crucial information is lacking regarding both its origin and prognosis. We aimed to characterize the course of cholestasis and factors contributing to it in patients with deficiency due to pituitary stalk interruption syndrome (PSIS).

**Methods:**

We conducted a retrospective single-center, case-cohort study including 16 patients with PSIS diagnosed before one year of age. We collected clinical and biological parameters from medical records and compared the characteristics of the endocrine syndrome in PSIS patients with and without cholestasis.

**Results:**

5/16 patients had cholestasis, all with a neonatal onset and multiple hypothalamic-pituitary deficiency. Patients with cholestasis presented with lower Apgar score and higher rate of ophthalmic malformations: 3/5 vs 1/11, p = 0.03 and 5/5 vs 4/11, p = 0.02, respectively. The plasma cortisol level was strongly decreased in patients with cholestasis: 12.4 ng/mL (8–15 ng/mL) vs 79.4 ng/mL (10–210 ng/mL), p = 0.04. Cholestasis resolved within 9 months following hormone supplementation. No development of chronic liver disease was observed during a median follow-up of 9.4 years (range, 1.3–13.3 years).

**Conclusions:**

Cholestasis is a frequent symptom at presentation of PSIS during the neonatal period that may help earlier diagnosis and that indicates a profound cortisol deficiency.

## Introduction

Pituitary stalk interruption syndrome (PSIS) is a rare disorder characterized by the combination of specific findings in magnetic resonance imaging (MRI): interrupted pituitary stalk, absent or ectopic posterior pituitary and anterior pituitary hypoplasia[1]. When present, PSIS indicates a permanent hypothalamic-pituitary (HP) deficiency. Early detection is crucial since hormone deficiencies lead to both life-threatening recurrent acute events and to long-term disabling morbidities[2, 3] in the absence of hormone supplementation. Because of the heterogeneity in the clinical, biological and imaging presentations, recent efforts have focused on discriminating patients with multiple HP deficiencies from isolated GH deficiency[4]. Association of PSIS with other syndromes and/or malformations[5–7] and the occurrence of familial forms have led to the identification of gene mutations[8] and suggested that abnormal birth conditions, observed more frequently in PSIS patients with multiple HP deficiencies, were a consequence rather than the cause of the syndrome[4]. Seeking to identify factors that may help with early diagnosis, we were intrigued by the fact that some PSIS patients presented with a neonatal cholestasis[9], a condition that may also result from perinatal insults, *via* hepatic hypoxia or ischemia[10]. Evaluation of cholestasis as indicative of a particular clinical entity and identification of pathophysiological factors have not been performed in depth since available information mostly emerged from case reports[9, 11–25]. We had unique access to the, to our knowledge, largest cohort of PSIS patients reported to date. We wondered if the unique association of PSIS with cholestasis could reveal a useful diagnostic marker and if specificities in the pathophysiology of PSIS could explain neonatal cholestasis. We examined the course of cholestasis among PSIS patients and compared the characteristics of the endocrine syndrome between patients with and without cholestasis, by retrospectively analyzing data collected from 16 patients diagnosed before one year of age.

## Patients and Methods

### Ethics Statement

The protocol was approved by the Ethical Review Committee “Comité de Protection des Personnes Ile de France III”. All clinical investigations were conducted according to the principals expressed in the Declaration of Helsinki. Written informed consent was obtained from the childrens’ parents and included in their hospital medical record. No activity in addition to the routine patient care was performed due to the study. The authors had no direct interaction with study patients, except for their medical follow-up performed by R. Brauner. Patient information was anonymized by the medical secretary prior to analysis.

### Patients and study design

This retrospective, single-center, case-cohort study was performed in 16 children (10 boys) out of a total of 89 PSIS patients monitored for HP deficiency by a senior pediatric endocrinologist (R. Brauner) in a university pediatric hospital over 32 years. To be included, the patients had to be diagnosed with PSIS before the age of 1 year. The threshold for age was decided considering that most cases of cholestasis with a neonatal onset are detected before 1 year of age and may evolve by successive flares. The patients were classified according to the presence or absence of cholestasis as defined below. All data were recorded by reviewing the clinical charts of patients included in the study.

### Methods

#### General clinical and demographic data

The history of each patient, including consanguinity and familial forms of disease, was recorded. Pre- and perinatal histories were reviewed. We recorded gestational age (<37 or >41 weeks), delivery by breech and/or caesarean section, the indications for the caesarean section, perinatal abnormalities (Apgar score <6 at 5 min and/or neonatal resuscitation), height and weight at birth, micropenis in boys, hypoglycemia and the nature of the presenting symptom. Intrauterine growth retardation was defined as weight or height at birth below the 3^rd^ percentile for gestational age. Micropenis was defined as a penis length of less than 30 mm. The presence or antecedence of cryptorchidism as well as other malformations were recorded.

#### Endocrine involvement

Hypoglycemia was defined as a blood glucose concentration below 3 mmol/L after 2 days of age. Pituitary height was evaluated using MRI as previously reported[1]. The criterion for diagnosing growth hormone (GH) deficiency was a GH peak response of less than 6.7 ng/mL after two pharmacological stimulation tests, excluding the response to GH-releasing hormone, or during spontaneous hypoglycemia. Plasma insulin-growth factor (IGF)-1 was measured in all the patients except 2 (N: 29–143 ng/mL at 0–2 years). Pituitary functions other than GH were evaluated by measuring basal blood cortisol at 08 am, free thyroxin (FT4) and prolactin. The normal limits were 12–28 pmol/L for plasma FT4, 5–25 μg/L for basal prolactin, except for neonates, who had higher concentrations. Adrenocorticotropic hormone (ACTH) deficiency was diagnosed as basal plasma cortisol values below 40 μg/L in neonates and below 80 μg/L in older children, as no increase during hypoglycemia, and as low ACTH. The combined luteinizing hormone (LH) and follicle-stimulating hormone (FSH) deficiency is difficult to diagnose in the prepubertal age, but we considered it as highly probable in boys with micropenis and patients with no increase in LH/FSH in the gonadotropin-releasing hormone (GnRH) test as infants or in those who reached pubertal age. Complete HP deficiency was diagnosed by deficiencies in GH, TSH, ACTH, and LH/FSH. The follow-up for each patient included measurements of plasma FT4 and cortisol concentrations every one to two years, if their concentrations had previously been normal, to diagnose delayed deficiencies. To ensure that results of pituitary hormone tests were comparable throughout the whole study period, each new immunoassay method was cross-correlated with the previous method.

#### Hepatobiliary involvement

Cholestasis was detected by the presence of jaundice and/or discolored stools and confirmed by measuring serum bile acid concentration. When patients presented with clinical signs of cholestasis, other causes of neonatal cholestasis were excluded with appropriate diagnostic procedures[10]. To evaluate liver function, the following parameters were measured: serum alanine aminotransferase activity (ALT), serum aspartate aminotransferase activity (AST), serum γ-glutamyl transpetidase activity (γ-GT), alpha-foetoprotein, vitamin A, 25-OH-vitamin D3, vitamin E, cholesterol and triglycerides, prothrombin time as well as coagulation factors II, V and VII+X. Outcome was evaluated during the follow-up by senior endocrinologist or hepatologist physicians and resolution of cholestasis was obtained when all the clinical and biological were normalized.

#### Statistical analysis

Quantitative results were expressed as the mean (range, minimum-maximum) and as percentages. Mann-Whitney U-tests were performed to detect differences of distributions between groups. To detect differences for qualitative variables, Fisher’s exact test was performed.

## Results

### Characteristics of the population

We identified 16/89 (18%) children diagnosed with PSIS before the age of 1. All patients had an ectopic posterior pituitary gland and a hypoplastic anterior pituitary gland with a pituitary stalk stated as absent (n = 11), interrupted (n = 4) or thin (n = 1) **(Table 1)**. No intra-uterine growth retardation, preterm or post-term birth, as well as consanguinity or familial cases were noticed in the medical charts. Median age for diagnosis was 0.2 years. Ten patients (63%) were delivered by cesarean section, including 4 in breech presentation, and 4 (25%) patients had low Apgar score. With the exception of cases 15 and 16, all had hypoglycemia. Among the 10 boys, 6 had microphallus and/or cryptorchidism. Additionally, 9 (56%) had ophthalmic malformations and 3 (19%) a malformation syndrome: a septal agenesia (case 1), a Fanconi syndrome (case 12) and a DiGeorge syndrome (case 16). Fourteen (88%) patients had multiple HP deficiency and 2 (12%) patients (cases 15 and 16) had an isolated GH deficiency.

**Table 1:** Characteristics of the patients.

Case	Age at	Cesarean	Breech	Apgar	Hypoglycemia	Clinical	Anterior	Pituitary	Pituitary	Genital	Ophthalmic	Cortisol	GH peak	IGF-1	FT4	LH	FSH
	diagnosis (years)	section	Presentation	score <6		Jaundice	pituitary length (mm)	Stalk aspect	posterior aspect	anomalies	Malformations	(ng/mL)	(ng/mL)	(ng/mL)	(pmol/L)	peak (U/L)	peak (U/L)
1^a^	0.0	Yes	No	No	Yes	No	Small	Interrupted	Ectopic	/	No	62	4.7	49	15.7	NA	NA
2	0.0	No	No	Yes	Yes	No	0	Absent	Ectopic	1^b^	No	12	3.8	NA	7	0.8	0.7
3	0.1	No	No	Yes	Yes	Yes	2.1	Absent	Ectopic	No	Optic nerve hypoplasia	15	5.1	3	7.4	0.27	0.3
4	0.1	Yes	No	No	Yes	Yes	Normal	Absent	Ectopic	1^b^	Astigmatism, myopia, strabismus	18	1.7	8	9	NA	NA
5	0.1	Yes	No	No	Yes	No	2.5	Absent	Ectopic	1^b^	No	10	1.8	10	10.8	NA	NA
6	0.1	Yes	Yes	No	Yes	No	NA	Interrupted	Ectopic	1^c^	Strabismus	26	2.5	33	7.6	0.9	<0.4
7	0.1	No	No	No	Yes	Yes	2	Absent	Ectopic	No	Optic nerve hypoplasia, strabismus	9	2.8	18	11.9	NA	NA
8	0.1	Yes	Yes	No	Yes	No	Small	Interrupted	Ectopic	/	No	38	2.1	19	9.7	NA	NA
9	0.2	Yes	Yes	Yes	Yes	Yes	NA	Absent	Ectopic	No	Astigmatism, hyperopia, strabismus	12	1.2	23	8.5	NA	NA
10	0.2	Yes	No	No	Yes	No	0	Absent	Ectopic	/	No	14	0.6	NA	7	<0.2	<0.42
11	0.2	Yes	Yes	Yes	Yes	Yes	2	Absent	Ectopic	/	No	8	2.8	3	8.1	NA	NA
12^a^	0.4	No	No	No	Yes	No	1	Absent	Ectopic	1^b^^,^^c^	Microphthalmia	210	2.3	14	12	3.9	3.9
13	0.8	No	No	No	Yes	No	0.5	Absent	Ectopic	/	No	130	2.8	57	11	4.8	6.4
14	0.9	Yes	No	No	Yes	No	Small	Absent	Ectopic	1^b^^,^^c^	Optic nerve hypoplasia and atrophia	16	2.0	12	8	14.5	2.4
15	1.0	No	No	No	No	No	3	Interrupted	Absent	No	Ptosis	205	2.8	14	13.1	0.27	0.3
16^a^	1.0	Yes	No	No	No	No	3	Thin	Ectopic	/	No	150	2.9	16	15	NA	NA

^a^: children with malformation syndrome

^b^: boys with micropenis

^c^: boys with cryptorchidism

NA: Not Assessed

/: not relevant (girls).

### Hepatobiliary history and risk factors

Jaundice was present in 5/16 (31%) patients and was one of the earliest clinical sign in addition to hypoglycemia **(Table 1)**. All the patients with cholestasis were first admitted in the Pediatric Hepatology Department of Hopital Bicetre; stools were discolored and the liver was enlarged in all 5 cases **(Table 2)** whereas the spleen was not palpable in any patient. These clinical features were not noticed for any of the other 11 patients. Presence of cholestasis was confirmed by the presence of an elevated serum bile acid concentration (mean: 373 μmol/L; range, 137–680 μmol/L; N<15 μmol/L). Mean total serum bilirubin concentration was 167 μmol/L (range, 84–351 μmol/L; N<17 μmol/L), mean serum conjugated bilirubin concentration was 133 μmol/L (N<7 μmol/L), mean serum γ-GT activity was 120 U/L (36–217 U/L; N<45 U/L), mean serum AST was 654 U/L (98–1525 U/L; N <50 U/L), and mean serum ALT was 288 U/L (45–630 U/L; N <45 U/L). Mean serum albumin concentration was 35 g/L (33–39, N: 38–45 g/L) and mean clotting factor V was 92% (58–100; N >80%). Mean serum cholesterol concentration was 3.09 mmol/L (2.32–4.13, N: 1.80–4.55 mmol/L), mean serum triglyceride concentration was 1.33 mmol/L (1.16–1.56, N: 0.35–1.20 mmol/L). Mean serum vitamin A, D and E concentrations were 98 μg/L (30–210, N: 170–370 μg/L), 19 ng/mL (12–30, N: 30–100 ng/mL), 1.98 mg/L (1.00–2.50, N: 7.0–15.0 mg/L), respectively **(Table 2)**. Measurement of prothrombin time and clotting factors showed vitamin K deficiency in one patient (case 4). No familial history of cholestasis was noticed in any of the 16 patients. The prevalence of low Apgar score and ophthalmic malformations was significantly greater in the patients with cholestasis than in those without: 3/5 (60%) vs 1/11 (9%), p = 0.03 and 5/5 (100%) vs 4/11 (36%), p = 0.02 **(Table 3).** Interestingly, while no statistically significant difference between the two groups for plasma GH peak, IGF-1 or FT4 levels could be found, the plasma cortisol level was profoundly decreased in patients with cholestasis: 12.4 ng/mL (8–15 ng/mL) vs 79.4 ng/mL (10–210 ng/mL), p = 0.04.

**Table 2:** Clinical and biological hepatobiliary characteristics of cases.

Case	Pale stools	Hepatomegaly	Serum bile acids (μmol/L)	T/C bili (μmol/L)	γ-GT(U/L)	AST (U/L)	ALT (U/L)	Albumin (g/L)	V (%)	Cholesterol (mmol/L)	Triglycerides (mmol/L)	Vitamin A (μg/L)	Vitamin D (ng/mL)	Vitamin E (mg/L)	α-foetoprotein (U/L)
3	Yes	Yes	680	188/160	36	1331	630	33	100	2.32	1.43	120	20	1.80	1091
4	Yes	Yes	407	351/251	217	1525	575	35	58	3.16	1.23	30	12	1.00	10300
7	Yes	Yes	137	101/97	185	188	134	37	100	4.13	1.56	210	30	2.20	7138
9	Yes	Yes	282	112/80	80	127	45	39	100	3.39	1.26	88	21	2.50	3940
11	Yes	Yes	361	84/77	84	98	57	33	100	2.43	1.16	41	13	2.39	4127

T/C bili: Total/Conjugated bilirubinemia; NA: Not Assessed

**Table 3:** Comparison between PSIS patients with or without cholestasis.

	All PSIS (n = 16)	PSIS with cholestasis (n = 5)	PSIS without cholestasis (n = 11)	P-value
Boys (%)	10 (63)	4 (80.0%)	6 (55%)	0.33
Age at diagnosis, months	0.55	0.14	0.33	0.52
Breech presentation	4 (25%)	2 (40.0%)	2 (18%)	0.35
Caesarean section	10 (63%)	3 (60.0%)	7 (64%)	0.89
**Low Apgar score (<6)**	**4 (25%)**	**3 (60.0%)**	**1 (9%)**	**0.03**
Hypoglycemia	15 (93%)	5 (100.0%)	10 (91%)	0.49
Micropenis (% boys)	5/10 (50%)	1/ 4 (25.0%)	4/6 (67%)	/
Cryptorchidism (% boys)	4/10 (40%)	0/4 (0.0%)	4/6 (67%)	**/**
Other syndromes	3 (19%)	0 (0.0%)	3/11 (27%)	/
**Ophthlamic malformations**	**9 (56%)**	**5 (100.0%)**	**4/11 (36%)**	**0.02**
Anterior pituitary height, mm	1.5 (n = 13)	2.0 (n = 3)	1.3 (n = 10)	0.41
Pituitary stalk				0.19
Absent	11 (69%)	5	6	
Interrupted	4 (25%)	0	4	
Thin	1 (6%)	0	1	
**Cortisol, ng/mL**	**58.4**	**12.4**	**79.4**	**0.04**
GH peak (ng/mL)	2.6	2.7	2,6	1.00
IGF-1, ng/mL	20.4 (n = 14)	11.0 (n = 4)	25.1 (n = 10)	0.10
Free T4, pmol/L	10.6	9.0	10.6	0.50
LH peak, U/L	3.21 (n = 8)	0.27 (n = 1)	3.62 (n = 7)	/
FSH peak, U/L	1.9 (n = 8)	0.30 (n = 1)	2.07 (n = 7)	/

### Outcome for children with cholestasis

**Fig 1:**
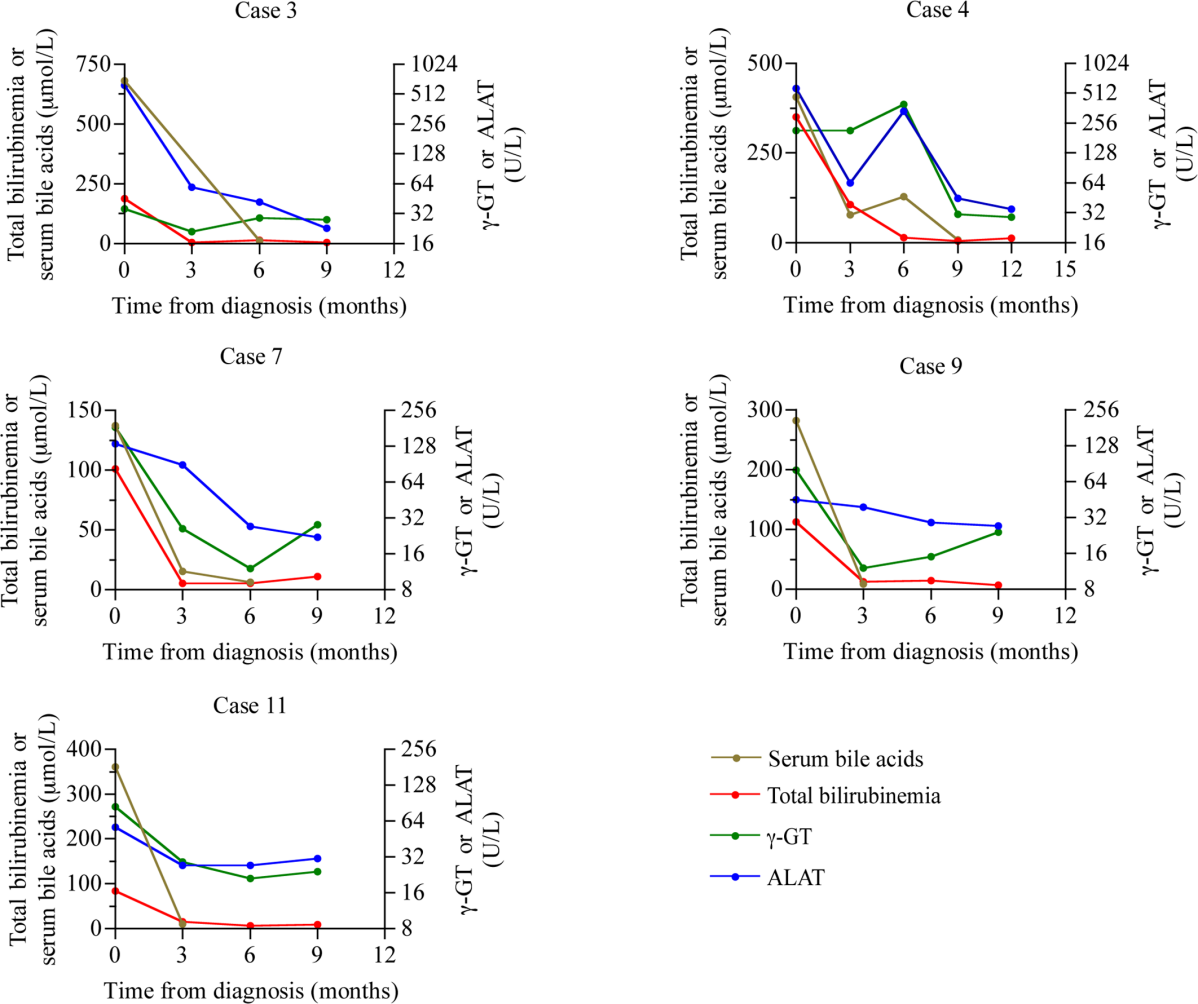
Evolution of serum bile acids, bilirubin, γ-GT and ALT levels with age (time from diagnosis) in the 5 PSIS patients with cholestasis.

Similar to the control group, patients with cholestasis were supplemented with deficient hormones with the exception of case 8, for whom GH supplementation was started 3 months after diagnosis due to the social situation of the parents. Additionally the 5 patients with clinical cholestasis were supplemented with fat-soluble vitamins A, D, E and K until the resolution of cholestasis; 3/5 patients were prescribed ursodeoxycholic acid. Clinical and biological parameters associated with cholestasis resolved in all instances. Stools were normally colored 3 months after diagnosis and the liver was still enlarged in one child until 9 months after diagnosis. No pruritus was reported during follow-up. Liver test results, including serum bile acid concentration, were normal for 3/5 patients 3 months after diagnosis and in all instances after 9 months **(Fig 1)**. After a median follow-up of 9.4 years (range, 1.3–13.3 years), no sign of chronic liver disease was reported. When looking for factors potentially associated with favorable outcome, we noticed that 2 patients (cases 3 and 9) among the 3 that had received ursodeoxycholic acid (cases 3, 7 and 9) had the most rapid normalization of liver tests.

## Discussion

We provide the first case-control study that allows for the identification of factors discriminating PSIS patients with or without cholestasis. We found that neonatal cholestasis is an underestimated feature of PSIS patients and confirmed the favorable outcome after hormone supplementation; our pioneer comparison with a group of PSIS patients without cholestasis strongly supports the role of cortisol deficiency in the onset of cholestasis.

### Prevalence of cholestasis in PSIS

With a prevalence of 5/16 (31%) in this series, we show that neonatal cholestasis is a frequent clinical feature of PSIS patients. Indeed, previsouly published information emerged from case reports or small series of clinically heterogeneous patients where the frequency of jaundice varied from 6 to 42%[15, 23, 26–28]. This substantial variation might be explained by differences in the nature of the deficient hormone axis and age at diagnosis. Furthermore, the cited studies did not discriminate between cholestatic and non-cholestatic jaundice. It is also conceivable that previous reports underestimated the frequency of cholestasis since a non-trained evaluator can easily miss subtle modifications in skin or stool color, especially when the magnitude of bilirubinemia is low.

### Course of liver disease

Little is known concerning both the management and outcome of cholestasis in the context of congenital HP deficiency. In rare cases the prognosis was remarkably poor, with the development of chronic liver disease and cirrhosis, leading to death[15, 17, 19, 23, 25]. However the vast majority of reports, including the most recent ones and our work, suggest a rapid and long-term resolution of cholestasis (median of 65 (42–150) days), after early hormone supplementation[11–14, 16, 18, 20–22, 24, 29]. None of the cited reports have described the consequences of biliary secretion dysfunction on vitamin absorption; we have shown that serum vitamin A, D and E levels were profoundly decreased in all tested patients with cholestasis and rapidly normalized with both vitamin supplementation and cholestasis resolution. We propose that all patients with hormone deficiency and cholestasis should be tested for vitamin levels and supplemented if required. This is of particular relevance when considering that one of our patients presented with a transient coagulopathy due to vitamin K deficiency (case 4) and that time to cholestasis resolution slightly varies from a series to another. Ursodeoxycholic acid is a widely used anticholestatic drug that contributes to accelerating recovery in the setting of transient neonatal cholestasis[10]. In the present series, ursodeoxycholic acid may have contributed to the faster resolution observed for two patients. We propose that ursodeoxycholic acid should be considered as adjuvant therapy, especially given the fact that it is well tolerated.

### Origin of cholestasis

While hormone deficiency is probably the strongest explanation for neonatal cholestasis in PSIS patients, the precise axis responsible is still a subject of debate: a review of clinical literature, which consists exclusively in case reports and series, is not conclusive. Analysis of data collected from our survey strongly correlates the presence of cholestasis with a profoundly collapsed plasma cortisol level, indicating that the adrenal axis might be responsible for liver involvement. This finding is also supported by the presence of cholestasis–even though it has been inconstantly reported—in patients suffering from isolated glucocorticoids deficiency[11, 30]. In addition, in most cases cholestasis rapidly decreases after cortisol supplementation is started, further suggesting that cortisol deficiency is responsible for cholestasis. This hypothesis is in line with experimental studies showing that adrenalectomy in rat leads to a reduction in biliary flow as a consequence of cortisol deficiency since cortisol supplementation was sufficient to correct the phenotype[31]. In 9 children with cholestasis due to hypopituitarism, liver biopsy analysis revealed a decreased expression of canalicular transport proteins (namely BSEP, MDR3, MRP2) involved in bile secretion[32]. Accordingly, glucocorticoids have been shown to regulate transcription of the genes encoding BSEP and MDR3 through different receptors (GR, PXR, FXR), directly or through the transcription factor C-terminal binding protein (CtBP)[33]. Other hormones, GH or/and IGF-1 or/and FT4, might participate to the onset and the persistence of cholestasis in PSIS, as supported by experimental studies: hypophysectomy in rats not only reduced bile acid synthesis and bile flow but also decreased bilirubin and bromosulfophtalein transport[34]. While GH was prominent in restoring a normal function, thyroid hormone had a potentiating role in the correction. These results are consistent with the molecular interaction of GH and thyroid hormone in the regulation of Mrp2[35], the canalicular transporter principally responsible for both bilirubin and bromosulfophtalein excretion. Contrasting with these findings, we did not identify any infant with isolated GH deficiency and suffering from cholestasis in this series, and IGF-1 and FT4 levels were not different between patients with and without cholestasis. Altogether, as suggested by Leblanc et al.[22] and also discussed by Gonç et al.[16], our results highlight cortisol deficiency as the precipitating factor for cholestasis in patients with PSIS rather than other pituitary hormones, even if we cannot exclude a potentiating role of GH or/and the TSH axis. However, due to the association with genital and ophthalmic anomalies, unidentified gene mutations might be an alternative explanation for neonatal cholestasis in PSIS patients. In the context of PSIS, perinatal hypoxia can be observed, likely due to adrenal deficiency or/and hypoglycemia and might contribute, at least in part, to cholestasis. Accordingly we observed a lower Apgar score in PSIS patients with cholestasis that might be the consequence of perinatal hypoxia, as described during transient neonatal cholestasis in which cholestasis is attributed to anoxo-ischemic injury of the liver[10].

## Conclusions

Cholestasis is a frequent symptom at presentation of PSIS during the neonatal period and associates with profound cortisol deficiency. Detecting cholestasis may help earlier diagnosis of HP deficiency especially when occurring after a breech presentation, or when associated with hypoglycemia, micropenis and cryptorchidism syndromes or with ophthalmic malformations. In this context, repeated evaluations of plasma cortisol, ACTH and GH concentrations during spontaneous hypoglycemia, as well as FT4 and IGF-1 concentrations should be measured. In case of low values, HP MRI will confirm HP deficiency. Early diagnosis of ACTH deficiency is crucial and has to be followed by hydrocortisone treatment in emergency settings. Parents and patients should be carefully and repeatedly informed about the necessity of: increasing the hydrocortisone dose during stress; switching to injections in the presence of any gastrointestinal symptoms or in the context of anesthesia procedures. Altogether these measures may reduce the delay in diagnosis and decrease both the morbidity and mortality. Ursodeoxycholic acid might accelerate the cholestasis resolution and prevent cirrhosis development.

## References

[1] Argyropoulou M, Perignon F, Brauner R, Brunelle F. Magnetic resonance imaging in the diagnosis of growth hormone deficiency. J Pediatr. 1992;120(6):886–91. Epub 1992/06/01. . 1593348 10.1016/s0022-3476(05)81955-9

[2] Mills JL, Schonberger LB, Wysowski DK, Brown P, Durako SJ, Cox C, et al Long-term mortality in the United States cohort of pituitary-derived growth hormone recipients. J Pediatr. 2004;144(4):430–6. Epub 2004/04/08. 10.1016/j.jpeds.2003.12.036 . 15069388

[3] Taback SP, Dean HJ. Mortality in Canadian children with growth hormone (GH) deficiency receiving GH therapy 1967–1992. The Canadian Growth Hormone Advisory Committee. J Clin Endocrinol Metab. 1996;81(5):1693–6. Epub 1996/05/01. 10.1210/jcem.81.5.8626817 . 8626817

[4] Pham LL, Lemaire P, Harroche A, Souberbielle JC, Brauner R. Pituitary stalk interruption syndrome in 53 postpubertal patients: factors influencing the heterogeneity of its presentation. PLoS One. 2013;8(1):e53189 Epub 2013/01/12. 10.1371/journal.pone.0053189 23308160 PMC3538767

[5] Dupuis-Girod S, Gluckman E, Souberbielle JC, Brauner R. Growth hormone deficiency caused by pituitary stalk interruption in Fanconi's anemia. J Pediatr. 2001;138(1):129–33. Epub 2001/01/10. 10.1067/mpd.2001.109200 . 11148528

[6] Leblanc T, Gluckman E, Brauner R. Growth hormone deficiency caused by pituitary stalk interruption in Diamond-Blackfan anemia. J Pediatr. 2003;142(3):358 Epub 2003/03/18. . 12640392 10.1067/mpd.2003.57

[7] Pinto G, Netchine I, Sobrier ML, Brunelle F, Souberbielle JC, Brauner R. Pituitary stalk interruption syndrome: a clinical-biological-genetic assessment of its pathogenesis. J Clin Endocrinol Metab. 1997;82(10):3450–4. Epub 1997/11/05. 10.1210/jcem.82.10.4295 . 9329385

[8] Reynaud R, Gueydan M, Saveanu A, Vallette-Kasic S, Enjalbert A, Brue T, et al Genetic screening of combined pituitary hormone deficiency: experience in 195 patients. J Clin Endocrinol Metab. 2006;91(9):3329–36. . 16735499 10.1210/jc.2005-2173

[9] Herman SP, Baggenstoss AH, Cloutier MD. Liver dysfunction and histologic abnormalities in neonatal hypopituitarism. J Pediatr. 1975;87(6 Pt 1):892–5. Epub 1975/12/01. . 1185390 10.1016/s0022-3476(75)80900-0

[10] Jacquemin E, Lykavieris P, Chaoui N, Hadchouel M, Bernard O. Transient neonatal cholestasis: origin and outcome. J Pediatr. 1998;133(4):563–7. Epub 1998/10/27. . 9787700 10.1016/s0022-3476(98)70070-8

[11] Al-Hussaini A, Almutairi A, Mursi A, Alghofely M, Asery A. Isolated cortisol deficiency: a rare cause of neonatal cholestasis. Saudi J Gastroenterol. 2012;18(5):339–41. Epub 2012/09/26. 10.4103/1319-3767.101137 23006463 PMC3500024

[12] Braslavsky D, Keselman A, Galoppo M, Lezama C, Chiesa A, Galoppo C, et al Neonatal cholestasis in congenital pituitary hormone deficiency and isolated hypocortisolism: characterization of liver dysfunction and follow-up. Arq Bras Endocrinol Metabol. 2011;55(8):622–7. Epub 2012/01/06. . 22218445 10.1590/s0004-27302011000800017

[13] DeSalvo D, Pohl JF, Wilson DP, Bryant W, Easley D, Greene J, et al Cholestasis secondary to panhypopituitarism in an infant. J Natl Med Assoc. 2008;100(3):342–4. . 18390029 10.1016/s0027-9684(15)31249-9

[14] Drop SL, Colle E, Guyda HJ. Hyperbilirubinaemia and idiopathic hypopituitarism in the newborn period. Acta Paediatr Scand. 1979;68(2):277–80. Epub 1979/03/01. . 419996 10.1111/j.1651-2227.1979.tb05003.x

[15] Ellaway CJ, Silinik M, Cowell CT, Gaskin KJ, Kamath KR, Dorney S, et al Cholestatic jaundice and congenital hypopituitarism. J Paediatr Child Health. 1995;31(1):51–3. Epub 1995/02/01. . 7748692 10.1111/j.1440-1754.1995.tb02914.x

[16] Gonc EN, Kandemir N, Andiran N, Ozon A, Yordam N. Cholestatic hepatitis as a result of severe cortisol deficiency in early infancy: report of two cases and review of literature. Turk J Pediatr. 2006;48(4):376–9. Epub 2007/02/13. . 17290578

[17] Hodges S, Buckler JM. Neonatal cholestasis and hypopituitarism. Arch Dis Child. 1984;59(12):1200 Epub 1984/12/01. 6524957 10.1136/adc.59.12.1200 PMC1628915

[18] Karnsakul W, Sawathiparnich P, Nimkarn S, Likitmaskul S, Santiprabhob J, Aanpreung P. Anterior pituitary hormone effects on hepatic functions in infants with congenital hypopituitarism. Ann Hepatol. 2007;6(2):97–103. Epub 2007/05/24. . 17519832

[19] Kaufman FR, Costin G, Thomas DW, Sinatra FR, Roe TF, Neustein HB. Neonatal cholestasis and hypopituitarism. Arch Dis Child. 1984;59(8):787–9. Epub 1984/08/01. 6476883 10.1136/adc.59.8.787 PMC1628640

[20] Lacy DE, Nathavitharana KA, Tarlow MJ. Neonatal hepatitis and congenital insensitivity to adrenocorticotropin (ACTH). J Pediatr Gastroenterol Nutr. 1993;17(4):438–40. Epub 1993/11/01. . 8145102 10.1097/00005176-199311000-00018

[21] Lanes R, Blanchette V, Edwin C, Zahka K, Lee PA, Pakula LC, et al Congenital hypopituitarism and conjugated hyperbilirubinemia in two infants. Am J Dis Child. 1978;132(9):926–8. Epub 1978/09/01. . 685913 10.1001/archpedi.1978.02120340102022

[22] Leblanc A, Odievre M, Hadchouel M, Gendrel D, Chaussain JL, Rappaport R. Neonatal cholestasis and hypoglycemia: possible role of cortisol deficiency. J Pediatr. 1981;99(4):577–80. Epub 1981/10/01. . 7277099 10.1016/s0022-3476(81)80260-0

[23] Lovinger RD, Kaplan SL, Grumbach MM. Congenital hypopituitarism associated with neonatal hypoglycemia and microphallus: four cases secondary to hypothalamic hormone deficiencies. J Pediatr. 1975;87(6 Pt 2):1171–81. Epub 1975/12/01. . 1185416 10.1016/s0022-3476(75)80132-6

[24] Machado MK, Bernardini A, Giachetto G. [Neonatal cholestasis and hypoglycemia like form of congenital hypopituitarism presentation]. Arch Argent Pediatr. 2011;109(3):e59–61. Epub 2011/06/11. 10.1590/S0325-00752011000300014 . 21660379

[25] Spray CH, McKiernan P, Waldron KE, Shaw N, Kirk J, Kelly DA. Investigation and outcome of neonatal hepatitis in infants with hypopituitarism. Acta Paediatr. 2000;89(8):951–4. Epub 2000/09/08. . 10976837 10.1080/080352500750043413

[26] Arrigo T, De Luca F, Maghnie M, Blandino A, Lombardo F, Messina MF, et al Relationships between neuroradiological and clinical features in apparently idiopathic hypopituitarism. Eur J Endocrinol. 1998;139(1):84–8. Epub 1998/08/14. . 9703383 10.1530/eje.0.1390084

[27] Pena-Almazan S, Buchlis J, Miller S, Shine B, MacGillivray M. Linear growth characteristics of congenitally GH-deficient infants from birth to one year of age. J Clin Endocrinol Metab. 2001;86(12):5691–4. Epub 2001/12/12. 10.1210/jcem.86.12.8068 . 11739421

[28] Huet F, Carel JC, Nivelon JL, Chaussain JL. Long-term results of GH therapy in GH-deficient children treated before 1 year of age. Eur J Endocrinol. 1999;140(1):29–34. Epub 1999/02/26. . 10037248 10.1530/eje.0.1400029

[29] Binder G, Martin DD, Kanther I, Schwarze CP, Ranke MB. The course of neonatal cholestasis in congenital combined pituitary hormone deficiency. J Pediatr Endocrinol Metab. 2007;20(6):695–702. Epub 2007/08/01. . 17663294 10.1515/jpem.2007.20.6.695

[30] Vallette-Kasic S, Brue T, Pulichino AM, Gueydan M, Barlier A, David M, et al Congenital isolated adrenocorticotropin deficiency: an underestimated cause of neonatal death, explained by TPIT gene mutations. J Clin Endocrinol Metab. 2005;90(3):1323–31. Epub 2004/12/23. 10.1210/jc.2004-1300 . 15613420

[31] Bauman JW Jr., Chang BS, Hall FR. The effects of adrenalectomy and hypophysectomy on bile flow in the rat. Acta Endocrinol (Copenh). 1966;52(3):404–8. Epub 1966/07/01. . 6013150 10.1530/acta.0.0520404

[32] Grammatikopoulos A, Knisely AJ, Hinds R, Byrne J, Thompson RJ, Hadzic N. Hepatocellular expression of canalicular transport proteins in infants with hypopituitarism. J Pediatr Gastroenterol Nutr. 2006;42(E4-E5). 10.1016/j.jpeds.2018.05.009 29935878

[33] Lu Y, Zhang Z, Xiong X, Wang X, Li J, Shi G, et al Glucocorticoids promote hepatic cholestasis in mice by inhibiting the transcriptional activity of the farnesoid X receptor. Gastroenterology. 2012;143(6):1630–40 e8. Epub 2012/08/28. 10.1053/j.gastro.2012.08.029 . 22922423

[34] Gartner LM, Arias IM. Hormonal control of hepatic bilirubin transport and conjugation. Am J Physiol. 1972;222(5):1091–9. Epub 1972/05/01. . 5022368 10.1152/ajplegacy.1972.222.5.1091

[35] Simon FR, Iwahashi M, Hu LJ, Qadri I, Arias IM, Ortiz D, et al Hormonal regulation of hepatic multidrug resistance-associated protein 2 (Abcc2) primarily involves the pattern of growth hormone secretion. Am J Physiol Gastrointest Liver Physiol. 2006;290(4):G595–608. Epub 2006/03/16. 10.1152/ajpgi.00240.2005 . 16537972

